# Supplementary figures and images for: Deep mutational scans of XBB.1.5 and BQ.1.1 reveal ongoing epistatic drift during SARS-CoV-2 evolution
Source: PLoS Pathog. 2023 Dec 29;19(12):e1011901. doi: 10.1371/journal.ppat.1011901 (PMC10783747; doi:10.1371/journal.ppat.1011901)

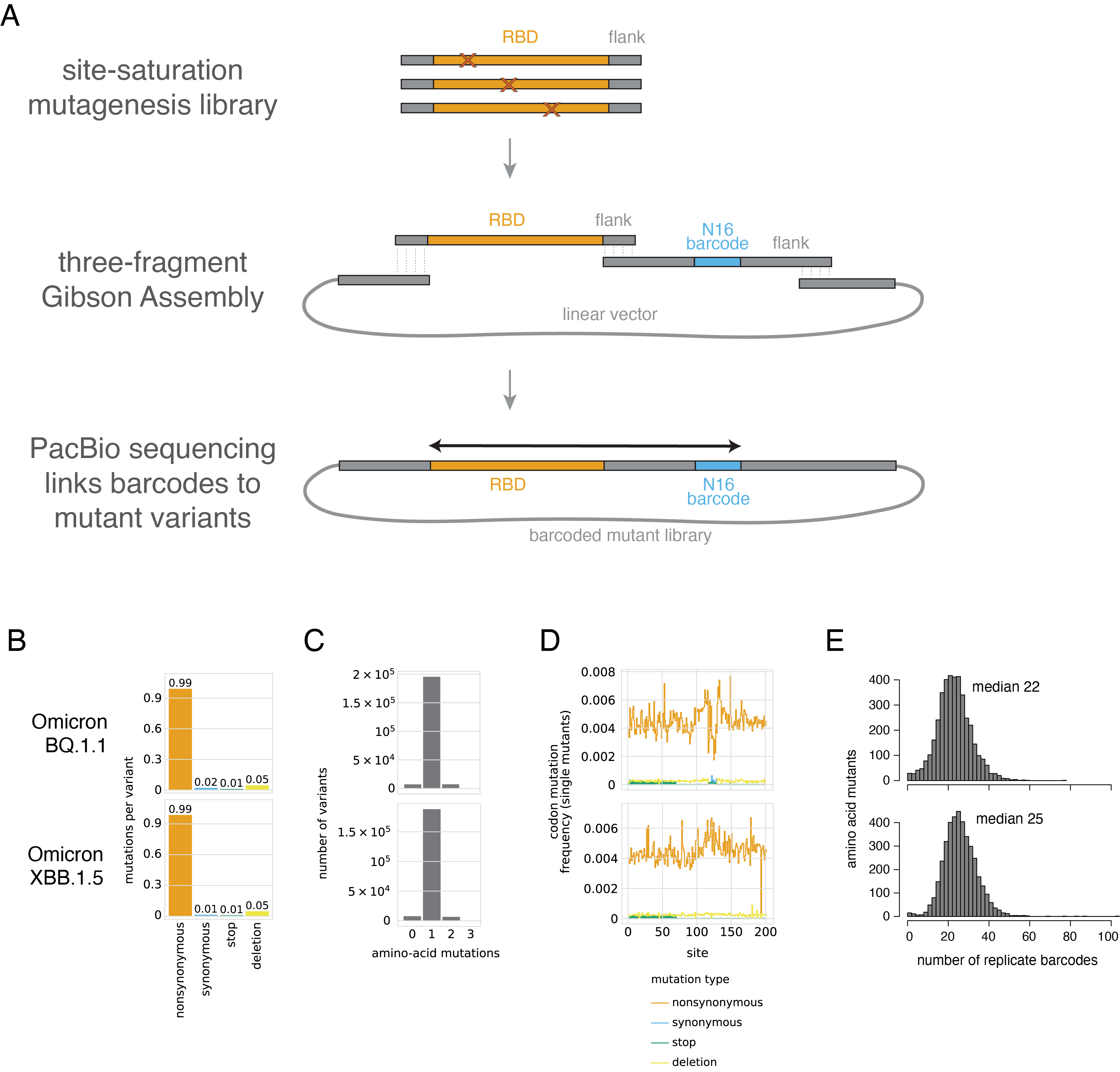

Supplement: S1 Fig — (A) Scheme for the generation of Omicron BQ.1.1 and XBB.1.5 mutant libraries. Site saturation mutagenesis oligonucleotide libraries were constructed by Twist Bioscience with constant flank sequences and cloned via three-fragment Gibson Assembly to create libraries of barcoded mutant variants. PacBio sequencing of the barcoded mutant library plasmid was used to create barcode:variant lookup tables, enabling subsequent Illumina sequencing of barcode fragments in experimental partitions to generate mutant phenotypes. (B-E) For pooled duplicate BQ.1.1 (top) and XBB.1.5 (bottom) libraries, we show (B) the per-variant rate of mutation types, (C) the distribution of number of amino acid mutants per barcoded variant, (D) the mutation rate of each type along each site in the RBD sequence, and (E) the distribution of total number of barcodes that were averaged for each amino acid mutant in the final ACE2-binding score. (PNG) [file ppat.1011901.s001.png]

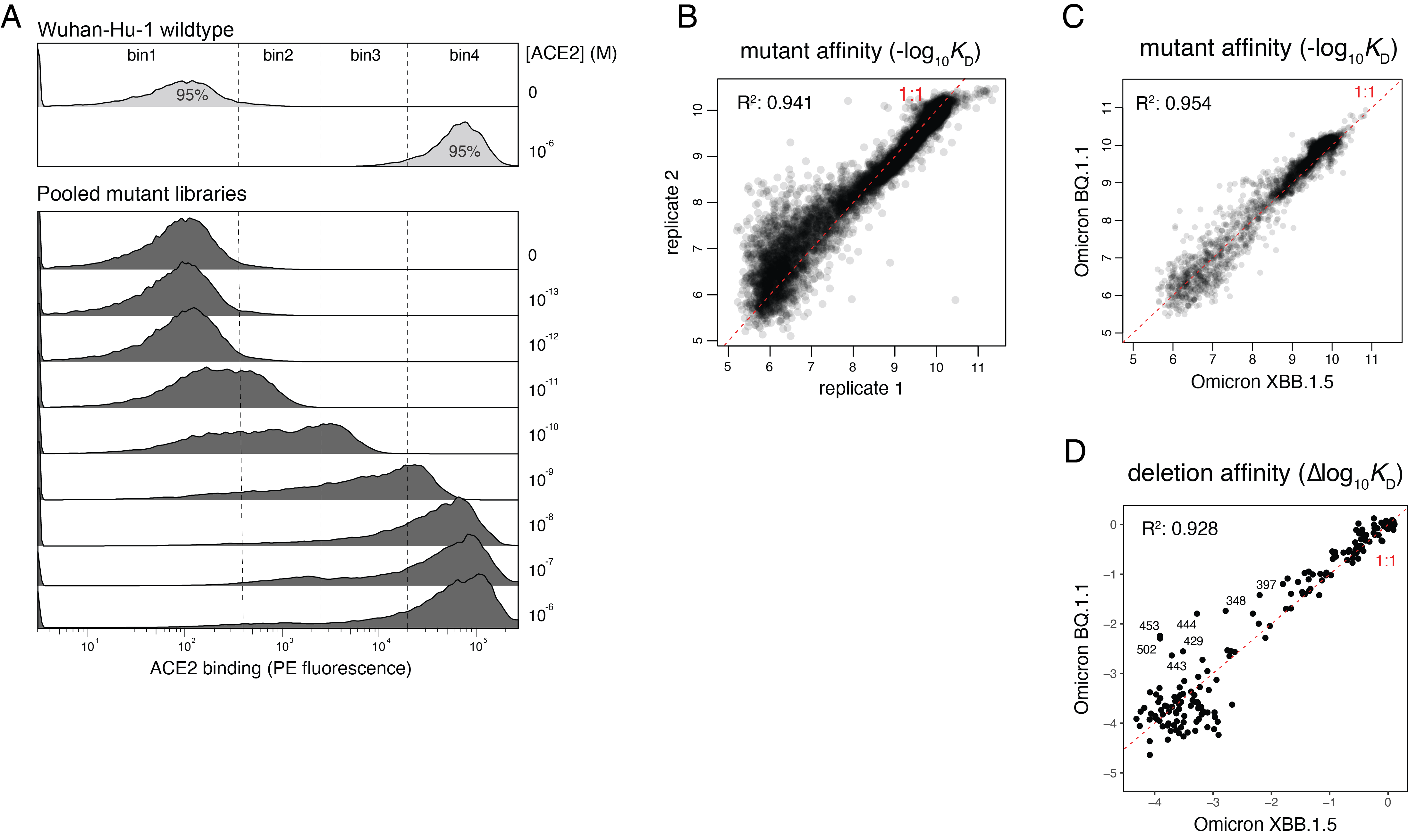

Supplement: S2 Fig — (A) Representative FACS binning scheme for ACE2-binding titration assays. Bins of PE fluorescence (ACE2 binding) were drawn on cells pre-selected on FSC/SSC and FITC(RBD)/FSC plots to isolate single RBD-positive cells. At each ACE2 concentration, >10 million cells were collected in total across the four bins. Post-sort cells were sequenced to enable deconvolution of the titration of each barcoded library variant in parallel. (B) Correlation in mutant ACE2-binding affinities in experimental duplicates (independently barcoded and assayed mutant libraries). Red dashed line indicates the 1:1 linear line. (C, D) Relationship between amino acid mutant (C) and deletion (D) affinities in the Omicron BQ.1.1 versus XBB.1.5 backgrounds. (PNG) [file ppat.1011901.s002.png]

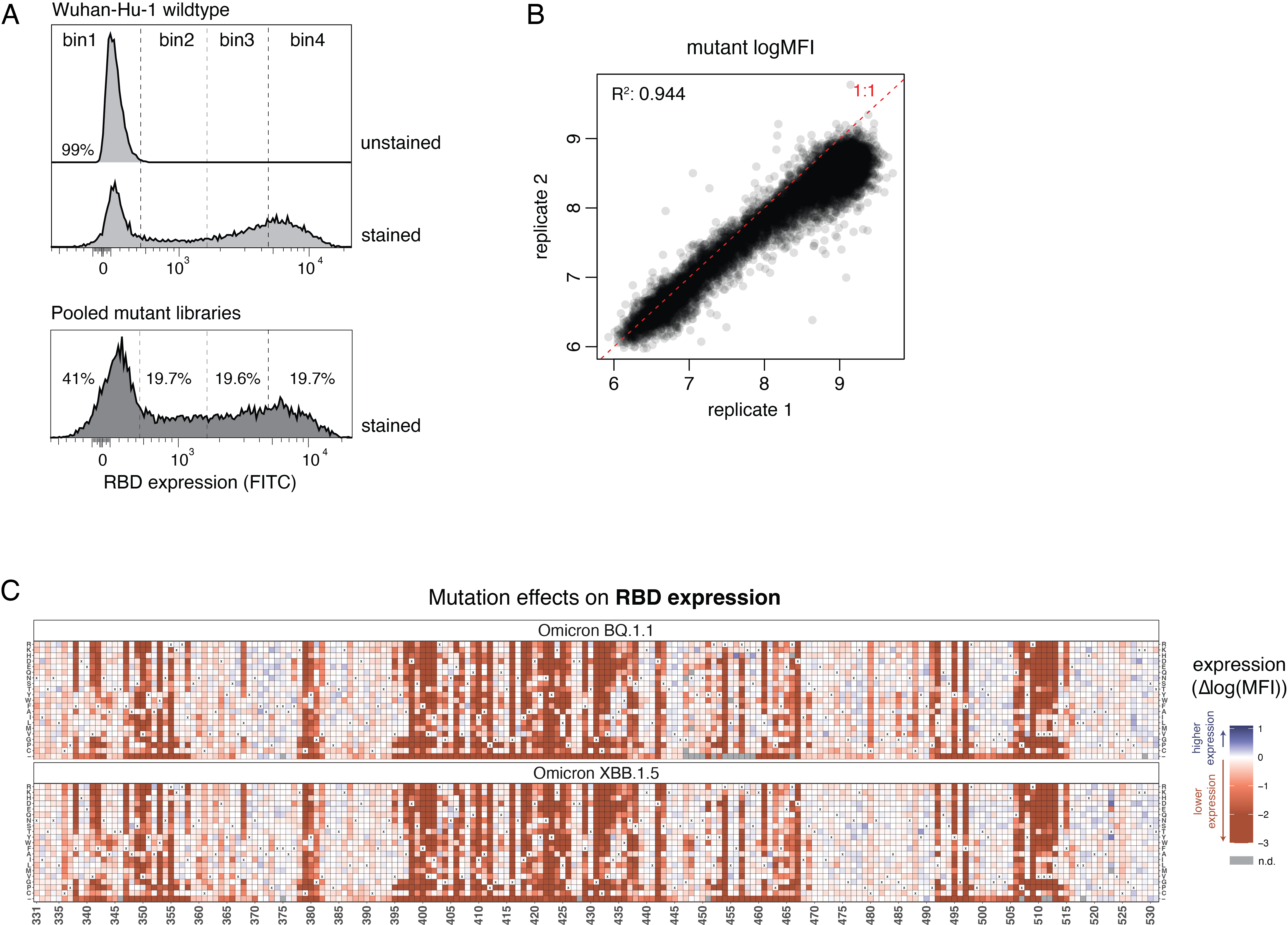

Supplement: S3 Fig — (A) Representative FACS scheme used for RBD expression deep mutational scans. Bins of FITC fluorescence (RBD expression) were drawn on cells pre-selected on FSC/SSC plots to isolate single cells. Cells were collected across the four bins and sequenced to identify the distribution of each library variant across bins and calculation of per-variant expression (mean fluorescence intensity, MFI). (B) Correlation in mutant RBD expression measurements in experimental duplicates (independently barcoded and assayed mutant libraries). Red dashed line represents the 1:1 linear line. (C) Heatmaps illustrate the impacts of all mutations in the BQ.1.1 and XBB.1.5 RBDs on RBD expression. (PNG) [file ppat.1011901.s003.png]

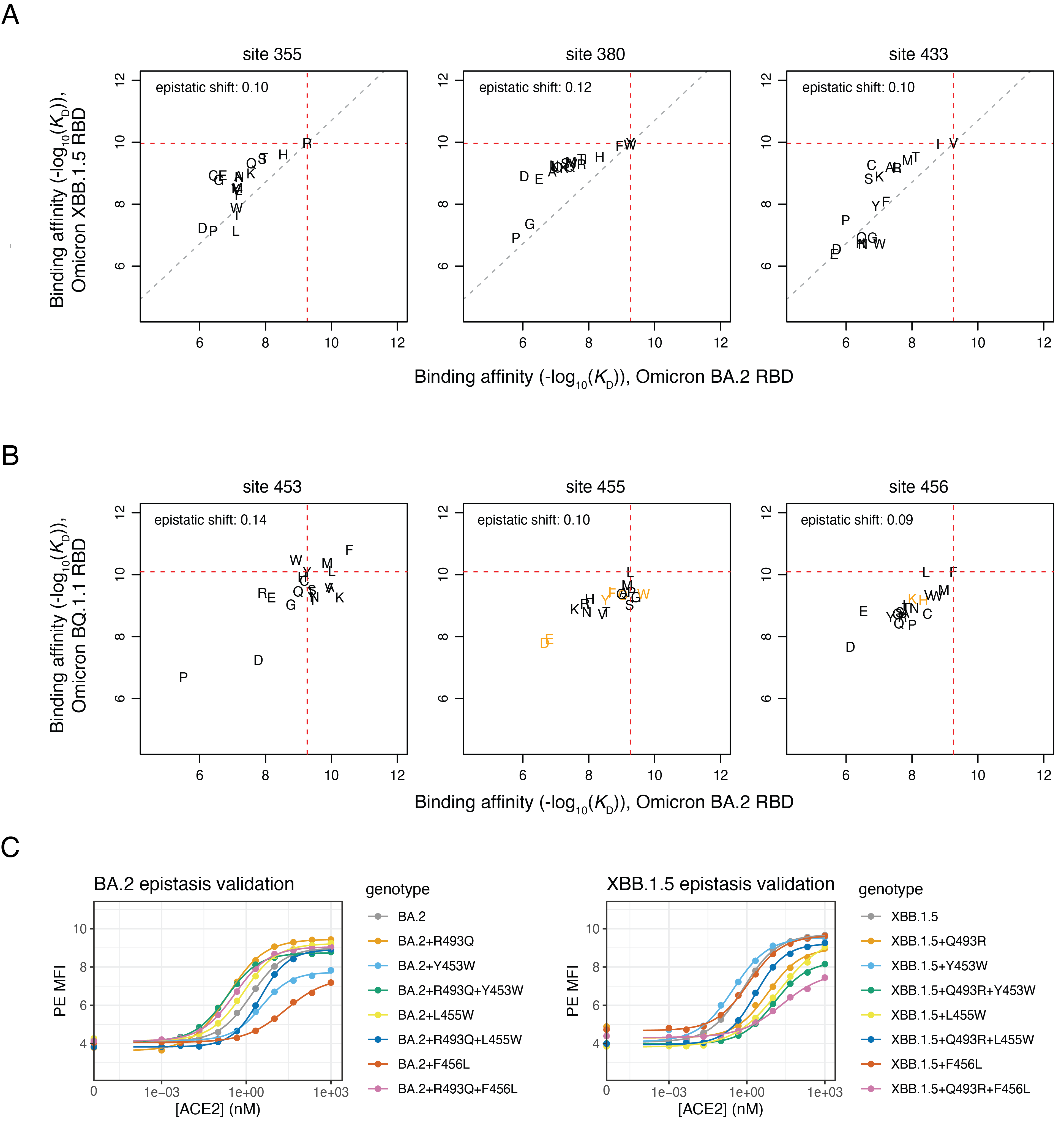

Supplement: S4 Fig — (A) Scatterplots of per-mutant affinities as in Fig 3B, illustrating nonspecific epistasis with impacts of mutations at buried core positions between XBB.1.5 and BA.2, with modestly affinity-decreasing mutations showing amplified effects in the BA.2 background. (B) Scatterplots of per-mutant affinities at sites 453, 455, 456 as shown in Fig 3B but for BQ.1.1 data. Orange letters indicate mutants that were assayed with fewer than three replicate barcodes in one of the two backgrounds; these mutants are excluded from the computation of the epistatic shift metric to reduce impacts of noise on the aggregate epistatic shift metric. (C) Representative binding curves from one of the three replicates of epistasis validation illustrated in Fig 3D. Yeast-displayed RBD mutants were expressed, incubated across a concentration series of monomeric human ACE2, and PE fluorescence (ACE2 binding) was determined via flow cytometry. Standard hill curves were fit to infer the midpoint EC50 binding constant. (PNG) [file ppat.1011901.s004.png]
