# Supplementary material for: Deep mutational scans of XBB.1.5 and BQ.1.1 reveal ongoing epistatic drift during SARS-CoV-2 evolution
Source: PLoS Pathog. 2023 Dec 29;19(12):e1011901. doi: 10.1371/journal.ppat.1011901 (PMC10783747; doi:10.1371/journal.ppat.1011901)
Supplement: Attachment — Submitted filename: reviewer_response.docx [file ppat.1011901.s006.docx]

Reviewer #1:

**Part I - Summary**

The manuscript by Taylor and Starr uses their established deep mutational scanning technique to analyze the ongoing epistatic drift in the background of XBB.1.5 and BQ.1.1 lineages. Overall I think this is an important study that is well performed and well written. My comments are mostly designed to make the manuscript easier for the reader.

**Part II – Major Issues: Key Experiments Required for Acceptance**

Reviewer #1: Figure 1a. I appreciate why the authors did not want to list all of the mutations for each lineage but the figure is going to mislead a lot of readers because they aren’t going to see the note. The authors need to find a way to visually demonstrate that the BA.2 mutations are still in the BQ/XBB. Perhaps extending the lines from the mutations in BA.2. downward.

We have modified this figure and the associated legend description to include dashed black lines on the BQ.1.1 and XBB.1.5 cartoons that propagate the WH1 -> BA.2 substitutions. We agree this makes it more clear to the reader that we are focusing the labels on the *additional* substitutions since BA.2 while still illustrating the continued accrual of substitutions that define the newer Omicron lineages.

Figure 1b. This is the meat of the manuscript, but it would be impossible to read in printed form (some people still print papers for reading). Recommend separating into two and making it a full-page figure.

We agree about the difficulty of representing deep mutational scanning datasets within figure size limits of traditional print publication. For that reason, we create websites that host the underlying data within an interactive heatmap visualization that makes it easier to view the results at per-mutation resolution, whereas the print heatmaps are best at showing patterns from a birds-eye view.

With regards to the print manuscript, we will defer to the journal editors on the best path forward for display of this large figure. Does PLOS Pathogens support “horizontally” oriented figures? If so, we can rotate this figure 90 degrees and compress the y-scale to fit it on a single page in the horizontal orientation, which will enable larger labels and heatmap squares for this figure.

Figure 2F is a problem. Without some more careful curating this is subfigure is only an illustration of where sequence dropouts are most likely to occur. I did a few spot checks of the sequences and noticed a few patterns. 1) the deletions were mostly not isolated but part of bigger deletions which are almost certainly sequencing artifacts. For instance, over 80% of the sequences with 482DEL (the most prevalent on their chart) also had 483DEL. 2) the sequences with the deletions mostly came from a handful of sequencing facilities. For instance, Canary islands make up less than 0.1% of all sequences in GISAID, but managed to generate 5-10% of the sequences with deletions (from the ones I checked). One partial solution to this would be to look for the sequences that have discrete deletions (I.E., search for sequences lacking one AA but not lacking the two flanking AAs). This isn’t perfect, but would get rid of most of the crap and it would be more of an ‘apples to apples’ comparison with the manuscript deletions. Alternatively they could restrict it to lineages with the same deletion more than once in the same background, but that would be a lot harder to compute. The subfigure is unacceptable in its current form.

We completely agree about the importance of curation when it comes to diagnosing deletions within GISAID sequences, as we likewise noted that deletions are frequently part of larger dropouts that are likely to be artefactual as the reviewer suggests. To ameliorate this issue, in the original analysis as described in the Methods we only began our alignment including spikes between 1260 and 1276 amino acids in length, and we further excluded from the deletion counting any RBD sequence that had more than four codons aligned as gaps across the RBD sequence (i.e. no more than four codons of consecutive and/or non-consecutive deletions). To address the specific example given by the reviewer, one recent accession from the Canary Islands is GISAID accession EPI_ISL_18209172, which has >>>4 deletions in the RBD and so was excluded in our original analysis. Our original QC method (which included additional exclusions as described in the Methods) eliminated 39% of the GISIAD accessions from analysis.

Nonetheless, we agree with the overall concern. First, we updated this analysis with a newer spike sequence set from GISAID as of November 11, 2023, incorporating newly reported sequences since our original analysis performed on sequences as of July, 2023. Besides replicating our original analysis excluding any RBD sequence with >4 gaps, we also repeated the analysis with a more stringent requirement, eliminating any RBD sequence with more than one single-codon gap. Results were qualitatively similar with either analysis method (see plots below for reference). Nonetheless, for enhanced stringency of QC we adopted the newer, more stringent method of only including RBD sequences in the alignment containing a single codon deletion (or full-length), which cleans up the number of apparent deletions of modest frequency with severely detrimental effects on ACE2-binding affinity.

Last, although these details of QC were previously described in the Methods, we also added a brief description of our QC in the main text so that other readers do not likewise believe we are including obviously pathological sequences such as EPI_ISL_18209172 in the analysis.

**Part III – Minor Issues: Editorial and Data Presentation Modifications**

Reviewer #1: Line 61-62. “in part to its ability to balance strength of ACE2 binding with immune escape via the beneficial F486P mutation”. Could the authors elaborate on what they mean by this. In their predictor F486P is not predicted to enhance ACE2 binding of BA.2 (the parent of XBB), but it is predicted to enhance binding of BQ.1.1, which never acquired it.

It is true that our measurements (not predictions) show that F486 is typically better for ACE2 binding than P486. I was using “beneficial” as a shorthand for what is expected to be overall a positive fitness gain due to two counteracting forces: mutations to F486 are thought to escape from a subset of neutralizing antibodies, but most of the single-nucleotide changes from the ancestral F486 codon have modest-to-large ACE2 binding deficits. Therefore, although F486 mutations have been sampled repeatedly (especially F486S, F486V) due to the drive for antibody escape, they were constrained by these deleterious impacts on ACE2 binding. P486 is typically the second-best amino acid for ACE2 binding in our measurements, and so would be best posed to balance the drive to escape F486-directed antibodies while suffering the least deficit in ACE2 binding of available mutations at the site. That is thought to be the reason why F486P was such an important contributor to XBB.1.5’s success. However, this is not the focus of the present manuscript and it’s a bit of a long explanation, which is why I originally lumped this information into the more concise description of F486P’s ability to “balance strength of ACE2 binding with immune escape”, underlying its likely beneficial effect on overall fitness. Rather than expand this explanation into a longer and more heavy-handed explanation, we have removed the word “beneficial” so as to reduce confusion between fitness and ACE2 binding (which is just one of many molecular determinants of ‘fitness’), so that the focus of the sentence is on the balance between immune escape and ACE2.

Line 139, reference Fig 1B. With the microprint I was initially waiting for the figure with the deletion data not realizing it was one of the lines in 1B.

We have fixed the subpanel designation and added a note to this figure reference to point the reader toward the bottom row of the heatmaps.

Line 171. Period missing.

We have corrected this typo.

Line 200. Confusing way of stating.

We have updated the language so as to not assume the reader knows what “sign epistasis” means.

Line 315. What’s are epistatic shits?

We have corrected this amusing typo and confirmed it is not present elsewhere.

Reviewer #2:

**Part I - Summary**

Taylor and Starr have performed deep mutational scans of XBB.1.5 RBD and BQ.1.1 RBD. The mutant libraries included single amino acid deletion, which is innovative and generated informative results. Epistatic shifts in both XBB.1.5 RBD and BQ.1.1 RBD were identified. The authors have further shown that the amino acid difference between XBB.1.5 and BQ.1.1 at position 493 modulated the effects of Y453W, L455W, and F456L on ACE2-binding. The analysis of epistatic drift at the end showed the importance of positions 498 and 501 in determining the epistatic landscape. Overall, this study is very nicely done and provides important insights into the SARS-CoV-2 evolution. I only have minor comments.

**Part II – Major Issues: Key Experiments Required for Acceptance**

None

**Part III – Minor Issues: Editorial and Data Presentation Modifications**

Reviewer #2: 1. Lines 34-36: “… the spike receptor-binding domain (RBD) has shown a particularly high rate of sequence evolution due to its role in binding ACE2 receptor to enable cell entry [2,3] …” It is unclear to me how the evolution of SARS-CoV-2 RBD is driven by its role in ACE2 binding.

The sentence that is abbreviated in this question is saying that the high rate of evolution is associated with both its role in ACE2 binding and antibody escape. Throughout the pandemic, we have seen clear selection both for mutations that influence ACE2 binding but not immune escape (e.g., N501Y, Q498R, R493Q) as well as those that impact immune escape (E484K, F486S/V/P, K444T, etc.), often with small detrimental effects on ACE2 binding. The subsequent sentence states our model for why ACE2 binding also contributes to evolution in the RBD, as mutations that escape antibodies often suffer mild or moderate deficits in ACE2 binding which drives compensatory mutations to restore ACE2 binding. We have added a citation to our prior manuscript here which shows in more detail how mutations like N501Y and Q498R that enhance ACE2 binding were important for Omicron’s evolution.

2. Lines 202-203: “Instead, these sites show greater sensitization to deleterious mutational effects in the BA.2 background (Supplemental Figure 4A).” It is a bit unclear to me how this conclusion can be drawn based on Supplemental Figure 4A. Additional explanation will be helpful. Maybe adding a gray dashed line indicating the additive (non-epistatic) expectation, just like Figure 3B, will also help.

We have added the suggested gray line as well as more description in the associated figure legend to clarify the trend that is shown in these scatterplots.

3. Lines 268-269: “… as well as the divergent human-ACE2-utilizing SARS-CoV-1 RBD …” Reference needed.

We have added the reference to our recent preprint that reports these data.

4. Lines 303-304: “… early fixation of Q498 substitutions would have instead driven SARS-CoV-2 closer to SARS-CoV-1 in the epistatic landscape.” Do the authors think that any aromatic amino acid substitutions (F, Y, W) at position 498 would similarly drive SARS-CoV-2 closer to SARS-CoV-1?

This is a very interesting question, and I appreciate it in helping us be more precise in our manuscript. For the purposes of this manuscript, we modified this statement to be more specific speculation about Q498Y, the exact SARS-CoV-1 state as it pertains to this statement. It is interesting to think in the future about whether Q498W would reveal other epistatic perturbations -- I would expect there to be different perturbations for Q498W versus e.g. Q498Y due to its different steric properties, but it would be very fun to explore this in the future with full combinatorial variation among the 20 possible amino acids at positions 501 and 498 simultaneously.
